# 18333 Utilizing community engagement approaches in translational research

**DOI:** 10.1017/cts.2021.595

**Published:** 2021-03-30

**Authors:** Keyonna M. King, Paul Estabrooks, Tatiana Tchouankam, Heidi Keeler, David Palm, Kenya Love, Christian I.J. Minter, Shinobu Watanabe-Galloway, Sean Navarrette, Maria Teel-Williams

**Affiliations:** University of Nebraska Medical Center

## Abstract

ABSTRACT IMPACT: Leverage community engagement to continue moving translational science and research forward. OBJECTIVES/GOALS: Engaging community in translational research improves innovation and speeds the movement of evidence into practice. Yet, it is unclear how community is engaged across the translational research spectrum or the degree of community-engagement used. We conducted a scoping review to fill this gap. METHODS/STUDY POPULATION: We used the PRISMA model search strategy with a range of databases (e.g., PubMed/Medline, Scopus) to identify articles published between January 2008 and November 2018 (n=167) and eliminated studies that did not use any level of community-engagement (n=102). Studies were coded for translational stage-corresponding to T0 (basic science), T1 (basic science to clinical research in humans; n=6), T2 (clinical efficacy and effectiveness research, n=45), T3 (dissemination and implementation research, n=95), and T4 (population health, n=21) as well as the degree of community engagement from least to most intensive (i.e., outreach, consultation, involvement, collaboration, shared leadership). RESULTS/ANTICIPATED RESULTS: The final number of eligible articles was 65. There was a relatively balanced distribution across levels of community engagement across articles (i.e., outreach, n=14; consultation, n=13; involvement, n=7; collaboration, n=15; shared leadership, n=16). Within these articles, the depth of community engagement varied with higher engagement typically occurring at later stages of translational research (T3 and T4), but more specifically in the dissemination and implementation science stage (T3). However, shared leadership, the most intensive form of engagement, was found in T2, T3, and T4 studies suggesting the value of community-engagement across the translational research spectrum. DISCUSSION/SIGNIFICANCE OF FINDINGS: A strong understanding of how various levels of community engagement are used in translational research, and the outcomes they produce, may to expedite the translation of knowledge into practice and enable practice-based needs to inform policy.

